# Controversy: the blanking period after atrial fibrillation ablation is needed and should be maintained

**DOI:** 10.1093/europace/euaf203

**Published:** 2025-09-01

**Authors:** Helmut Pürerfellner, Stylianos Tzeis, John Mandrola

**Affiliations:** Ordensklinikum Linz Elisabethinen, Department of Cardiology, Fadingerstr. 1, A-4010 Linz, Austria; Department of Cardiology, Mitera Hospital, Athens, Greece; Cardiac Electrophysiology, Baptist Health, Louisville, KY, USA

**Keywords:** Ablation, Atrial fibrillation, Blanking period, Recurrences, Pulsed field ablation

## Abstract

The concept of a post-ablation ‘blanking period’ (typically 90 days) has long served as a clinical and regulatory buffer during which atrial arrhythmia recurrences are not considered indicative of procedural failure. This period was originally justified by transient electrophysiological instability due to oedema, inflammation, and autonomic fluctuations. However, recent data—particularly in the era of continuous rhythm monitoring and novel energy sources such as pulsed field ablation—have challenged the biological and prognostic neutrality of early recurrences. Several studies now suggest that arrhythmia episodes within the blanking period, especially beyond the first 30 days, may strongly predict long-term recurrence, thereby questioning the utility of maintaining a fixed observational window. This EP Europace Controversy article presents opposing viewpoints on the necessity of the blanking period in modern practice. Dr Stelios Tzeis argues in favour of preserving the blanking period, citing its role in minimizing unnecessary re-intervention and ensuring standardized trial endpoints. Conversely, Dr John Mandrola advocates for shortening or abandoning the blanking period, proposing a more individualized approach guided by arrhythmia timing, symptom burden, and ablation modality. The introduction, authored by Prof. Helmut Pürerfellner, sets the stage by reviewing the historical rationale, emerging evidence, and practical implications of this ongoing debate. As ablation techniques evolve and data accrue, reassessing the blanking period becomes essential. This Controversy aims to inform clinical practice, future trial design, and shared decision-making by juxtaposing two expert perspectives on a timely and consequential topic in atrial fibrillation management.

## Graphical Abstract

**Graphical Abstract:**
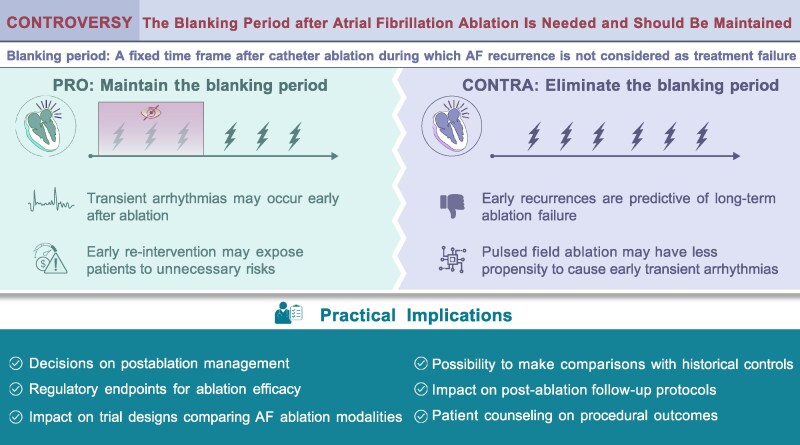

## Figures and Tables

**Figure 1:**
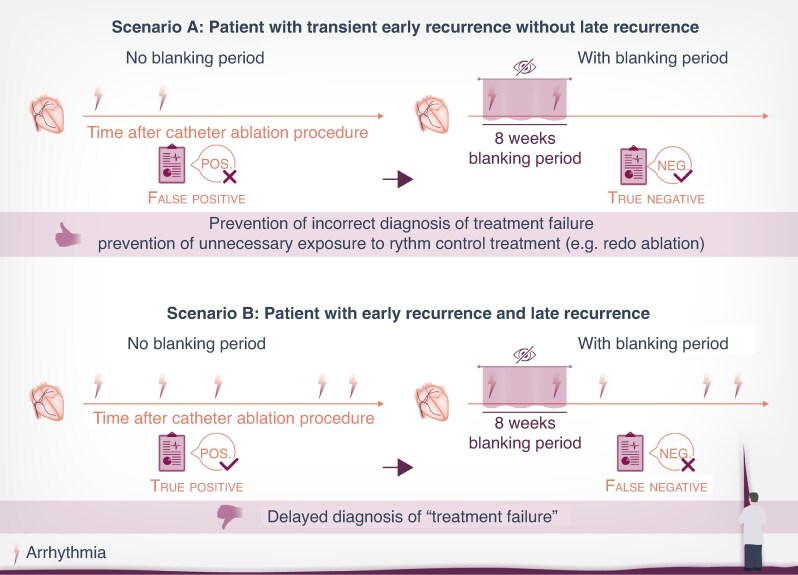
Scenarios of outcome reclassification following catheter ablation, depending on the implementation of a blanking period or not.

**Table 1:** Clinical trials assessing the incidence and prognostic significance of early arrhythmia recurrences following pulsed field ablation of atrial fibrillation

Trial—year	Type of catheter	Number of patients	Time window of ER	Monitoring protocol	% of pts with ER	Total FU duration	% of pts with ER without LR
Plank *et al.*/2023^26^	Pentaspline catheter, FARAPULSE, Boston Scientific	231 (55% PAF—45% PersAF)	90 days	12-lead ECG and 24–72 h Holter monitoring at 6 and 12 mo	19.9%	12 mo	45.8%
admIRE/2025^27^	Variable loop catheter, Varipulse, Biosense Webster	169 (100% PAF)	90 days	TTM (weekly for 1–5 mo, monthly from 6 to 12 mo and if symptomatic), 12-lead ECG (3, 6, and 12 mo), and 24 h Holter monitoring (6 and 12 mo)	18.3%	12 mo	29%
Bisignani *et al.*/2025^28^	Pentaspline catheter, FARAPULSE, Boston Scientific	620 (75% PAF—25% PersAF)	90 days	Not prespecified (combination of 12-lead ECG, 24 h Holter monitoring, symptom-triggered monitoring, or CIED interrogation when applicable)	7.1%	12 mo (mean FU)	45.5%
Mohanty *et al.*/2025^29^	Pentaspline catheter, FARAPULSE, Boston Scientific	337 (57% PAF—43% PersAF)	90 days	Event recorder (at least three transmissions per week and if symptomatic for the first 5 mo), 12-lead ECG, and 7-day Holter monitoring (1, 6, and 12 mo)	15.7%	12 mo	13.2%
PULSED-AF/2025^14^	PulseSelect System, Medtronic	294 (52% PAF—48% PersAF)	90 days	TTM (weekly and if symptomatic), 12-lead ECG (3, 6, and 12 mo), and 24 h Holter monitoring (6 and 12 mo)	27.1% (PAF) 31.6% (PersAF)	12 mo	29.4% (PAF) 34.3% (PersAF)

CIED, cardiac implantable electronic device; ECG, electrocardiogram; ER, early recurrence; FU, follow-up; mo, months; LR, late recurrence; PAF, paroxysmal atrial fibrillation; PersAF, persistent atrial fibrillation; TTM, transtelephonic monitoring.

## Supplementary Material

euaf156_Supplementary_Data

## Introduction

https://orcid.org/0000-0002-8965-8495PürerfellnerHelmut
Ordensklinikum Linz Elisabethinen, Linz, Austria

‘Living is easy with eyes closed misunderstanding all you see’ (Lennon/McCartney, Strawberry Fields Forever)

The landscape of atrial fibrillation (AF) ablation has undergone a transformation in the last decade. With increasing procedural volumes, improved outcomes, and the recent advent of novel energy sources such as pulsed field ablation (PFA), electrophysiologists are increasingly confronted with new clinical realities that challenge traditional paradigms. One such paradigm is the blanking period—a fixed time frame following catheter ablation, typically set at 90 days, during which recurrence of atrial arrhythmias is not considered a failure of the procedure.^1,2^

The blanking period has long been defended as a necessary buffer zone to allow for transient electrophysiological instability, stemming from tissue oedema, inflammation, autonomic imbalance, and evolving lesion maturation.^3^ It was formalized in early clinical trial protocols and incorporated into successive iterations of consensus statements. Recurrences during the blanking period—commonly referred to as early recurrence of atrial tachyarrhythmias (ERAT)—have historically been viewed as benign, provided that patients remained in sinus rhythm after the 3-month mark.^1,3^

In addition to recurrence timing, recent evidence has shown that AF burden—the total duration or percentage of time spent in AF—may be an even stronger predictor of long-term outcomes than timing alone. The European Society of Cardiology Council on Stroke and EHRA recently highlighted AF burden as a clinically meaningful endpoint that can better stratify recurrence risk and guide follow-up strategies.^4,5^ This perspective supports individualized, burden-based follow-up rather than a rigid temporal cut-off, though caution is warranted to avoid premature re-intervention based solely on early burden measurements.

Multiple recent studies have called the assumption of blanking period neutrality. Across various ablation modalities, including radiofrequency, cryoablation, and PFA, data suggest that ERAT may not be transient in nature but rather predictive of long-term procedural failure.^6–8^ In particular, a growing body of evidence shows that the timing of recurrence within the blanking period matters: ERAT within the first 30 days may indeed reflect reversible injury or temporary triggers, but episodes occurring beyond the first month may herald true reconnection or incomplete lesion formation.^7,9^ These findings, when combined with AF burden analysis, offer a more nuanced picture of recurrence risk.

This evolving understanding has prompted some to advocate for a shortened blanking period, especially in light of new technologies that offer sharper lesion demarcation, reduced collateral damage, and more durable pulmonary vein isolation. In 2024, an international consensus document endorsed this change, recommending a shortened 60-day blanking period following thermal ablation techniques and suggesting that data from PFA may support even shorter time frames, possibly eliminating the concept altogether for specific patient groups and ablation methods.^10^

Proponents of the traditional blanking period argue that transient recurrences do exist and that early re-intervention based on premature arrhythmia episodes may expose patients to unnecessary risks, procedural complications, and psychological distress. They caution that variability in inflammation, lesion resolution, and individual healing processes warrants a conservative, uniform window of observation. Moreover, from a trial design perspective, maintaining a consistent blanking period allows for standardized endpoints and comparability across studies.^1,3^ Shortening or abolishing the blanking period could complicate direct comparison with legacy data and disrupt longitudinal benchmarking of ablation outcomes.

On the other hand, critics of the conventional model suggest that ignoring early recurrence may delay timely re-intervention, especially in high-risk patients with persistent symptoms, large atria, or significant comorbidities. Furthermore, in the era of continuous rhythm monitoring, we now have the tools to detect even brief arrhythmia episodes, offering the opportunity to personalize follow-up and adapt therapy dynamically. Several recent trials have shown that patients with ERAT have a two- to four-fold increased risk of late recurrence, suggesting a strong prognostic signal that should not be disregarded.^7,9,11^

Pulsed field ablation has added another layer of complexity to this discussion. Due to its non-thermal mechanism, ultra-fast lesion formation, and minimal inflammatory response, PFA may offer a procedural substrate with less ‘noise’ during the early post-ablation phase. Emerging registries and observational studies suggest that early arrhythmia recurrence after PFA is not only uncommon but also often indicative of durable failure, challenging the notion that a 90-day buffer is necessary.^12,13^

The implications of this controversy extend beyond semantics. They affect:

- Clinical practice, as physicians must decide whether to reassure, escalate therapy, or repeat ablation based on early post-procedural episodes.
- Regulatory frameworks, as endpoints for ablation efficacy continue to rely on blanking-period-adjusted recurrence definitions.
- Trial design, as new trials test ablation strategies, energy sources, and early vs. late rhythm intervention.
- And most importantly, patient counselling, where clarity and consistency in defining procedural success or failure are critical to shared decision-making. Conflicting interpretations of procedural success can trigger anxiety and confusion in patients thereby impairing their quality of life.

Should the blanking period be abolished altogether? Should it be tailored to the ablation technology, the timing of recurrence, or the patient’s clinical profile? Should we distinguish between asymptomatic and symptomatic episodes, or between isolated AF vs. organized atrial tachycardias? These are the questions that electrophysiologists must now grapple with as we move towards more individualized, evidence-driven management of AF patients.

This Controversy article brings together two opposing but equally well-grounded viewpoints: On one side, a defence of the traditional blanking period—a tool rooted in biological plausibility and clinical pragmatism—and on the other side, a case for its modification or elimination, based on emerging evidence, real-world practice, and modern monitoring capabilities. Both perspectives will be informed by the latest literature and the authors’ own clinical experience in managing AF patients across the spectrum of complexity.

As we debate this issue, the goal remains unchanged: to provide safe, effective, and timely care for our patients taking into account not only recurrence metrics but also patient-centred endpoints such as quality of life, healthcare utilization, and psychological burden. Whether the blanking period still serves that goal—or stands in its way—remains the subject of this timely discussion.

### Data availability

No new data have been acquired for this paper.

## Pro

https://orcid.org/0000-0002-2007-1928TzeisStylianos
Department of Cardiology, Mitera Hospital, Greece

Catheter ablation is a well-established treatment for atrial fibrillation. It aims to alleviate arrhythmia-related symptoms, reduce the progression to more advanced and impactful AF types, and in specific patient categories, improve outcome. This invasive treatment has earned evidence-based credentials and has been upgraded as the recommended first-line treatment option in patients with recurrent symptomatic paroxysmal atrial fibrillation.^1^ Having entered the era of PFA in the latest years, we have been experiencing a substantial enrichment of our armamentarium with novel energy sources, ablation catheters, and procedural workflows.^2,3^ However, despite the accumulating experience and the technological advancements in ablation tools, a substantial percentage of patients experiences arrhythmia recurrences. A recent meta-analysis found that more than half of patients experienced arrhythmia recurrence within 5 years after a single ablation, with ∼70% achieving freedom from recurrence after multiple procedures.^4^ The existing heterogeneity in reported efficacy rates largely stems from inconsistencies in the methodology and rigor of post-ablation rhythm monitoring.^5^

### Blanking period: rationale and optimal duration

Several pathophysiological processes may trigger arrhythmogenesis during the immediate post-ablation phase. These proarrhythmic mechanisms are related to ablation-mediated acute tissue injury and include oxidative stress, myocardial oedema, local and systemic inflammatory response, ischaemia, myocardial necrosis, autonomic nervous dysbalance, atrial stretch, and pericardial irritation.^6–9^ These proarrhythmic phenomena may lead to early post-ablation recurrences, and their incidence varies mainly depending on the type of implemented monitoring techniques.^9^ However, given the transient nature of these alterations, many patients who experience early arrhythmia recurrences do not develop late recurrences. According to a meta-analysis of 17 studies, 38% of patients with early recurrences remained free from late recurrence (95% confidence interval: 33–44%).^10^

It should be noted that all early arrhythmia recurrences do not have the same prognostic value for predicting late recurrence. Several parameters are indicative that some early post-ablation recurrences are not transient and are associated with a higher risk of procedural failure. Specifically, a greater number of early recurrences within the blanking period are associated with a higher risk of later atrial fibrillation (AF) recurrences. Patients experiencing multiple early recurrences within the initial 6 weeks following ablation demonstrated lower long-term ablation success rates compared to those with isolated or no early recurrences.^11^ Furthermore, AF burden during the blanking period, measured either by intermittent, smartphone-based ECG, or continuous monitoring is a strong and independent predictor of late arrhythmia recurrences after ablation.^12–14^ Studies indicate that each 1% rise in AF burden during the blanking period elevates the risk of late recurrence by roughly 4–5%.^14,15^ In addition, the timing of early recurrence is a crucial factor that influences long-term rhythm outcome. In general, the later the occurrence of an ‘early recurrence’ within the post-ablation blanking period, the greater the likelihood of late recurrence. This time-dependent increase in the predictive power of early recurrence has been replicated in several studies with diverse types of energy sources used during the index ablation procedure.^10–12^

This observation of ‘late post-ablation cure’ has supported the introduction of a blanking period. This time window aims to blind rhythm monitoring and efficacy assessment since early detected recurrences may be attributed to the abovementioned transient proarrhythmic phenomena and therefore may not predict long-term arrhythmia recurrence or treatment failure. The 2007 Consensus Statement on Catheter and Surgical Ablation of Atrial Fibrillation advised the adoption of a 90-day post-ablation blanking period.^16^ This recommendation was reiterated in the subsequent 2012 and 2017 versions of the consensus statement.^17,18^

The reclassification scenarios and the practical issues associated with implementing a blanking period in the post-ablation setting are presented in *Figure 1*. Consider a patient experiencing an early recurrence but remaining free of late recurrences. Without a blanking period, this transient early recurrence would be misinterpreted as treatment failure (false positive: occurrence of early recurrence without late recurrence). By masking rhythm monitoring during the initial post-ablation phase and disregarding early arrhythmia recurrences, these patients are correctly classified as true negatives, thus reducing the number of false positives. Preventing the mislabelling of treatment failure in this patient subgroup is crucial, to avoid exposure to unnecessary rhythm control therapy and even early redo ablation procedures. Early re-interventions may present procedural challenges, as residual oedema may impede effective lesion formation, especially during thermal ablation. Furthermore, from a patient-centred perspective, they have a substantial impact due to related psychological stress, logistical burdens, and procedural risks.

The trade-off of implementing a blanking period is depicted in scenario B of *Figure 1*. In a patient with both early and subsequent late recurrences (true positive), the blanking period leads to misclassification as a false negative (lack of early recurrence with occurrence of late recurrence). This scenario occurs when early arrhythmia recurrence is not due to a subsiding post-ablation proarrhythmic state but rather to a persistent (e.g. reconnected pulmonary vein) or potentially not-targeted (e.g. extra-pulmonary vein) arrhythmic trigger. However, from a clinical standpoint, this misclassification simply delays the diagnosis of treatment failure until a later recurrence occurs beyond the blanking period. This delay assumes no clinical sequelae or associated risk, provided the patient continues appropriate anticoagulation based on their thromboembolic risk. This minimal trade-off is arguably worthwhile to reduce false positive diagnoses, mislabelling of patients as treatment failures, unnecessary redo ablation procedures, associated procedural risks, and related costs.

Robust data support shortening the blanking period to less than the arbitrary selected 90-day duration. However, the critical question remains: what is the best and most clinically meaningful way to define its optimal length? Analyses of receiver operating characteristic (ROC) curves aiming to predict late recurrence from early recurrence have identified several cut-off points. A meta-analysis of 17 studies reported that the optimal blanking period duration is 4 weeks, having a specificity and positive predictive value of 76%.^10^ However, the major caveat of this type of statistical methodology is that it fails to incorporate clinical considerations and equally weighs positive and negative predictive values, overlooking that earlier blanking period cut-offs inherently increase false positive rates. A secondary analysis of the CIRCA-DOSE trial evaluated the prevalence and impact of early recurrences following catheter ablation using continuous cardiac rhythm monitoring. While the optimal discriminatory cut-off on the ROC curve was 19.5 days, more clinically relevant cut-offs with higher specificity occurred later (90% specificity at 40 days and 96% specificity at 60 days).^5^ Furthermore, another analysis of the same dataset demonstrated that blanking periods shorter than 8 weeks are hindered by the reclassification of transient self-limited arrhythmia episodes of limited clinical significance as treatment failures.^19^ In the 2024 expert consensus statement on catheter and surgical ablation of atrial fibrillation, the writing group agreed on shortening the blanking period to 8 weeks.^20^ In consistency with previously reported considerations, this decision prioritized reducing false positives, maintaining a high positive predictive value, and minimizing the misclassification of early recurrences that do not indicate treatment failure.

### Blanking period still matters in pulsed field ablation!

As previously reported, the cascade of mechanisms and processes initiated by ablation lesion creation may partially explain the occurrence of transient early arrhythmias. However, the tissue injury mechanisms related to thermal ablation (radiofrequency and cryoablation) differ significantly from those of non-thermal PFA. In the latter, the delivery of pulsed electric energy disrupts cell membrane integrity, leading to cell death via irreversible electroporation. Proponents of eliminating the blanking period argue that the distinctive features of PFA lesions may trigger a less proarrhythmic sequence of tissue reactions, thus reducing transient arrhythmogenesis during the post-ablation healing phase, questioning the need for incorporating a blanking period.

Nevertheless, several arguments still support the need to maintain a blanking period even in the PFA era. The mechanism of lesion formation in PFA, although different from the thermal modalities, is not devoid of proarrhythmic mechanisms. The temporal evolution of the lesion following pulsed electrical field delivery encompasses an inflammatory phase marked by inflammatory cell infiltration during the initial 2 weeks.^21^ Measurements of IL-6 in patients undergoing catheter ablation with the pentaspline catheter demonstrate that PFA elicits an inflammatory response. Notably, this inflammatory activation was numerically lower, although statistically non-significant, compared to radiofrequency ablation.^22^ However, the precise cascade of pathways initiated by PFA and its resultant proarrhythmic sequelae remains to be fully elucidated. Furthermore, generalizing the potential for transient proarrhythmia from one PFA system to another is prone to bias. This is due to the significant variations in treatment parameters and waveform configurations across PFA systems, which can result in diverse lesion morphologies, sometimes even encroaching upon the thermal ablation phase. Pericarditis is also a well-established trigger for atrial fibrillation.^23^ Animal studies support a direct causal link between pericarditis and atrial fibrillation, at least partially mediated by the release of proinflammatory cytokines.^24^ The potential for acute pericarditis, even at a very low rate, following PFA underscores the existence of a transient arrhythmogenic factor and supports the rationale for maintaining a blanking period.^25^

*Table 1* presents the clinical studies evaluating the prognostic significance of early arrhythmia recurrences after PFA. Direct comparison of the incidence of early recurrences across PFA studies, or with those using thermal energy, is flawed by non-standardized and highly variable monitoring protocols. Nevertheless, it is of clinical importance that a notable proportion of patients (ranging from 13 to 45%) experiencing early recurrence within the first 3 months post-PFA (across various catheter and ablation systems) remains free from subsequent arrhythmia relapse until the completion of the reported follow-up. Therefore, the phenomenon of delayed post-ablation arrhythmia suppression still holds true with pulsed field energy, reinforcing the original rationale for the blanking period. However, we have to acknowledge that field of PFA is still evolving. Given that most current data are observational or derived from secondary analyses, robust data from randomized controlled trials are needed to further shape and support pertinent recommendations.

In conclusion, many decisions in medicine hinge on perspective. Do we prioritize high specificity to avoid false positives or high sensitivity to catch every potential case? Is our guiding principle ‘safety first’ or ‘efficacy first’? In the same page, the dilemma of implementing or eliminating a blanking period stems from conflicting perspectives on optimal patient post-ablation monitoring and management. The argument for abrogating the blanking period comes at the cost of reclassifying patients with transient, self-limited, and clinically insignificant arrhythmia episodes as ‘treatment failures’. This is not merely an issue of incorrect labelling. This approach exposes a non-negligible percentage of patients to the risk of unnecessary therapy with an associated, albeit small, risk of complications. The alternative perspective of maintaining a blanking period delays the diagnosis of recurrence in a limited number of false negative cases. Our conscience can far more easily bear the burden of this relatively painless misclassification.

## Contra

https://orcid.org/0000-0001-5093-9946MandrolaJohn
Cardiac Electrophysiology, Baptist Health, Louisville, KY, USA

### Assesment of current knowledge

Merely having this debate about a blanking period after AF ablation exposes the massive uncertainty regarding the condition of AF. Electrophysiologists struggle with this topic because we cannot agree on the purpose of AF ablation. Is it to reduce electrical episodes of AF on a monitor? Is it to improve quality of life? Is it to reduce adverse outcomes, such as stroke and heart failure? Does ablation cure AF?

Atrial fibrillation differs from almost every other condition in cardiology. We understand acute myocardial infarction well. Therapies were tested in trials of days to months duration without blanking periods; survival is the clear treatment goal. Heart failure therapies too can be easily assessed: treatments should improve survival and reduce heart failure episodes. Angina is a simple supply–demand mismatch; therapies either relieve symptoms or extend exercise time, or not.

Irregular rapid electrical activity in the atrium does not fit into such a frame. Sometimes AF is a sign, manifestation, or sequela of other conditions, such as after surgery or infection. It can also be a form fruste of an atrial tachycardia from the pulmonary veins or it can be a late manifestation of atrial structural disease from cardiometabolic disorders. Not only do these potential causes of AF vary, but so do the type of presentations, ranging from asymptomatic to severe heart failure.

Ablation, however, is the same in every case. We routinely electrically isolate the pulmonary veins or add lines in a highly inferior attempt to mimic surgical maze. Mostly this is done because we can. Twenty years into ablation, we cannot come close to answering the common question from patients: how do you know where to ablate?

Since the cause(s) of AF, its presentations, and even the goals of ablation vary so much, there is a lack of consensus on the most appropriate post-ablation metrics. Unlike quality of life endpoints, which would require sham-controlled trials, AF episodes are easy to measure, so we record them and use them as a marker of success of therapy. The blanking period after ablation arose from the observation that transient early recurrences of AF episodes can occur. While these early recurrences increase the likelihood of late recurrences, they often dissipate never to recur.

Yet an electrical episode on a monitor is just a convenient surrogate. The health implications of an episode of AF on a monitor have always been unclear. In *CABANA*, for instance, ablation reduced AF episodes vs. medical therapy, but this reduction did not lead to improved hard clinical outcomes.^1^ The advent of always-on medical devices, such as smartwatches, with their ability to detect short-duration, subclinical AF episodes, have rendered the AF episode even less certain as a surrogate marker of ‘anything important’.

Very recent data add even more uncertainty regarding the clinical importance of an AF episode. The *ARTESIA* and *NOAH-AFNET 6* trials of oral anticoagulation in subclinical AF demonstrated that AF in 2025 can be a much lower risk phenomenon than it was in years past.^2,3^ Patients in these trials had high estimated stroke risk but extremely low untreated stroke rates.

### The arguments against the blanking period

My main argument against the blanking period is philosophical. We should oppose a blanking period because it fosters simplistic thinking. Recall that the roots of electrophysiology were not in the speed of pulmonary vein isolation, but in deep thinking about cardiac physiology. Do you think Gorden Moe, Ken Rosen, Dirk Durrer, and Mark Josephson would be worried about blanking periods after pulmonary vein isolation? The great thinkers of electrophysiology would be struggling to describe the pathophysiology of AF.

Inherent in the acceptance of a blanking period—of any duration—is the notion that AF on a monitor means something important. Over the past two decades, instead of striving to define the pathophysiology of AF, its varied manifestations, and what constitutes treatment success, including the placebo effect of ablation, we lean on convenience—the AF episode on a monitor. Amusing is when increasingly sensitive monitors find short duration AF after ablation in about half of patients, we argue for switching our outcome of choice to AF burden.

I realize that *Europace* readers want more than a philosophical argument. Clinicians must be pragmatists, so here are four pragmatic reasons to jettison the blanking period.

As Mohamed Ruzieh, Andrew Foy, and I *wrote* recently, patients cannot pause for a blanking period.^4^ If a patient has to seek medical care—either in the clinic or hospital—they do not dismiss it as nothing because it occurs a month after their procedure to ‘fix’ their problem. If you are in the clinic or hospital, you are not at work or on your bike.

The second pragmatic reason to stop the blanking period is how it distorts the evidentiary basis of efficacy and safety of procedural interventions.

Two examples outside of AF illustrate the danger of including blanking periods after procedures. In *PREVAIL*, the FDA-mandated regulatory trial of Watchman vs. warfarin, there were two co-primary endpoints—one with and one without a blanking period. In the first co-primary endpoint, stroke, systemic embolism, and cardiovascular death, Watchman failed to meet non-inferiority against warfarin.^5^ It was the second co-primary endpoint of stroke and systemic embolism that excluded the 7 days post-procedure (e.g. a blanking period) that established PREVAIL as a ‘positive’ trial. Left atrial appendage occlusion proponents sometimes cite reductions in *non–procedure*-related major bleeding. But without blanking out the procedure, major bleeding was *similar* in both the Watchman and warfarin arms.^6^

Another example of distortion with blanking periods came in the *ISCHEMIA* trial of delayed vs. immediate coronary angiography in patients with stable coronary artery disease and ischaemia.^7^ The composite primary endpoint included myocardial infarction (MI), which included *all causes* of myocardial infarction, even those associated with the revascularization procedure. Using this definition, there were no significant differences in the rate of MI between the two treatment arms. If ISCHEMIA had a blanking period wherein procedural MI was not included, the trial would have shown a significant reduction in MI (HR 0.67, 95% CI 0.53–0.83). A blanking period may have changed the interpretation of ISCHEMIA, much like it did with left atrial appendage closure.

While these examples are outside of AF, they clearly show how blanking periods could bias in favour of a procedure over a comparator. Imagine our AF ablation vs. drug trials without a blanking period. Surely the ablation advantage would be reduced.

The advent of PFA provides the third pragmatic reason for eliminating the blanking period. Unlike thermal ablation, PFA creates less inflammation and has less propensity to cause early recurrences of atrial tachyarrhythmia. The *SINGLE SHOT CHAMPION* trial of PFA vs. cryoballoon ablation found PFA superior for the suppression of AF episodes.^8^ The Kaplan–Meier curves separated almost immediately after the procedure and remained parallel thereafter. In other words, it was the elimination of the early recurrences that made most of the difference with PFA over thermal ablation. The blanking period minimized the superiority of PFA in this trial.

The final reason to eliminate the blanking period is that there was never an evidentiary basis for it. It began with expert opinion and has been carried forward out of convenience. The argument for blanking periods began when we decided to measure AF episodes as an endpoint. If we had a better understanding of the goals of AF ablation, outcomes such as quality of life, rates of stroke (on or off oral anticoagulation), or hospitalizations would have been better choices of endpoints. None of these clinical patient-centred endpoints would have required a blanking period.

I recognize that some may worry that not having a blanking period could lead to overtreatment of early recurrences of arrhythmia after ablation. This worry demeans the clinical skills of electrophysiologists. It assumes that we treat ECGs instead of patients. The fair assessment of treatment strategies in clinical trials, which means counting events no matter when they occur, and the wise treatment of individual patients are not mutually exclusive.

Better late than never; it is time to jettison the blanking period after AF ablation. The day of ablation should be time zero, as it is for patients.

### Data availability

All data are incorporated in the article.
