# Supplementary material for: Controversy: the blanking period after atrial fibrillation ablation is needed and should be maintained
Source: Europace. 2025 Sep 1;27(10):euaf203. doi: 10.1093/europace/euaf203 (PMC12503109; doi:10.1093/europace/euaf203)
Supplement: euaf156_Supplementary_Data [file euaf203_supplementary_data.docx]

**Editorial comment**

Thanks for this submission in the section "Controversies". According to journal's style and taking into account the specificities of "Controversies" please adopt in the submission (by modifying the title in the submision site" this version of the titlle "Controversy: the blanking period after atrial fibrillation ablation is needed and should be maintained". The other title  has characteristics typical of an editorial and not typical for a Pro/Con which require a single sentence to be approached with a Pro and a Contra.

Answer to comment: We have reviewed and revised the manuscript to ensure compliance with the journal’s Instructions to Authors. This includes text formatting, reference style, figure preparation, and structural adjustments. Furthernore, we have revised the title of the manuscript to "***Controversy: the blanking period after atrial fibrillation ablation is needed and should be maintained***".

Please improve the quality of the graphical abstract Please revise  the text according to Reviewers' notes.
Answer to comment: Thank you for your suggestion. We have created a new graphical abstract to summarize the key arguments from each side and the implications of the decision to maintain or abolish the blanking period.

Reviewer #1:

Confidential comments to Author:

This well-structured controversy piece offers an informative and timely discussion on the relevance of the blanking period (BP) following atrial fibrillation (AF) ablation. The topic is highly pertinent given the recent technological advancements in ablation, particularly pulsed field ablation (PFA), and the increasing focus on optimizing procedural outcomes and endpoints. The manuscript is framed by a balanced introduction by Prof. Helmut Pürerfellner, which provides historical context and presents the clinical dilemma objectively. The opposing perspectives from Dr. Stylianos Tzeis and Dr. John Mandrola are clearly articulated and supported by appropriate literature. Dr. Tzeis's inclusion of recent PFA trial data in tabular form is a valuable addition. However, several aspects merit revision or further elaboration to enhance clarity, consistency, and clinical utility.

We would like to thank the reviewer for the positive comments

General Comments

1.      Title Consistency

Two different titles are presented:

o       "The Blanking Period (BP) After Atrial Fibrillation Ablation: Essential Clinical Buffer or Obsolete Dogma?"
o       "Controversy: the blanking period after atrial fibrillation ablation is needed and should be maintained"

Answer to comment: Based on your suggestion we have maintained a consistent title throughout the manuscript and also included this in the graphical abstract "***Controversy: the blanking period after atrial fibrillation ablation is needed and should be maintained***"

2.      AF Burden vs Timing of Recurrence

The manuscript emphasizes the timing of AF recurrences within the BP; however, AF burden is a more clinically relevant predictor of long-term outcomes. This supports individualized, burden-based follow-up rather than a rigid temporal cut-off. The following points require elaboration:

- AF burden as a stronger predictor of recurrence than timing alone.
- The need for extended follow-up to reliably assess burden (without implying a fixed BP)
- Reablation decisions based on early burden should be addressed cautiously, especially considering risks of premature intervention.

Answer to the comments: Based on the reviewer’s suggestion we have expanded the Introduction to clarify that AF burden, as demonstrated in recent consensus and trial data, is often a stronger predictor of long-term recurrence than timing alone. We also stress that early reablation decisions based solely on burden require caution, particularly given procedural risks and transient post-ablation changes.

Furthermore, in the PRO section we have highlighted the value of AF burden (and not timing) during the BP as a predictor of late recurrence: *“Furthermore, AF burden during the blanking period, measured either by intermittent, smartphone-based ECG or continuous monitoring is a strong and independent predictor of late arrhythmia recurrences after ablation.(12-14) Studies indicate that each 1% rise in AF burden during the blanking period elevates the risk of late recurrence by roughly 4-5%.(14, 15).”*

3.      Reablation Risks and Patient-Centered Considerations

Early reintervention may be counterproductive due to:

o       Residual edema impairing lesion formation (especially with thermal energy).
o       The psychological burden and logistical challenges faced by patients undergoing unexpected early repeat procedures.

These patient-centered and procedural factors should be acknowledged explicitly.

Based on the reviewer’s comments we highlighted explicitly the caveats of early reinterventions in the PRO section: “*Preventing the mislabeling of treatment failure in this patient subgroup is crucial, to avoid exposure to unnecessary rhythm control therapy and even early redo ablation procedures. Early reinterventions may present procedural challenges, as residual edema may impede effective lesion formation, especially during thermal ablation. Furthermore, from a patient-centered perspective they have a substantial impact due to related psychological stress, logistical burdens, and procedural risks*.”

4.      Comparability with Historical Trials

Removal or modification of the BP complicates comparison with legacy trials that excluded early recurrences. This has important implications for:

- Meta-analyses.
- Regulatory endpoint harmonization.
- Longitudinal benchmarking of ablation outcomes.

We totally agree with the reviewer’s comment. The issue of comparability with historical trials was highlighted in the introduction of the initial version (Pürerfellner) where we stated that: “*Moreover, from a trial design perspective, maintaining a consistent BP allows for standardized endpoints and comparability across studies*“. In the revised manuscript we further emphasized this by adding: “*Shortening or abolishing the BP could complicate direct comparison with legacy data and disrupt longitudinal benchmarking of ablation outcomes*.

5.	 Colloquial Language in the Contra Section

Several phrases (e.g., "don't," "can't," "mindlessly," "soft thinking") are too informal for a scientific journal.
Please, replace them with formal equivalents to maintain academic tone.

Further to the reviewer’s comment we have replaced informal phrases (“don’t,” “can’t,” “mindlessly,” “soft thinking”) with more formal language while preserving the intended meaning. The term 'soft thinking' was replaced with “simpistic thinking”.

6.      Terminological Inaccuracies

- Page 17: "AF is a symptom of other conditions" → AF should be described as a sign or manifestation, not a symptom.
- Page 18: "My main argument against the blanking period, therefore, is philosophical." The use of "therefore" is unclear here. If the argument is philosophical, it should not be positioned as a logical consequence of preceding data.

We have made the following correction based on the reviewer’s suggestions: “*Sometimes AF is a sign, manifestation or sequela of other conditions, such as after surgery or infection.”*

7.      Graphical Abstracts and Visuals

o       Graphical Abstract 1: The point "Newer technologies enable more durable AF ablation" is not inherently a justification for eliminating the BP. In fact, more durable outcomes would predict fewer recurrences within the BP, reinforcing its diagnostic value.

Additional axes to consider:

AF burden as an alternative endpoint.

Historical data continuity.

Simplification of post-ablation follow-up protocols.

Further to your suggestion we have modified the graphical abstract. First, its artistic part has been prepared by a professional designer and contentwise we made the following modifications: (a) we have modified the point "*Newer technologies enable more durable AF ablation*" to “*Pulsed field ablation may have less propensity to cause early transient arrhythmias*” and (b) in the part of practical implications we included “*Possibility to make comparisons with historical controls*” and “*Impact on post-ablation follow-up protocols*”

o       Graphical Abstract 2: The figure quality is suboptimal, with distorted cardiac icons and blurred components—hallmarks of AI-generated visuals. It should be replaced with a professionally designed, publication-ready alternative.

Thank you for the comment. However, we would like to verify that Figure 2 “Scenarios of outcome reclassification following catheter ablation, depending on the implementation of a blanking period or not.” was created by a professional designer without the use of AI.

8.      Standardization of Terminology

Terms such as early recurrence, transient arrhythmia, false positives, and false negatives are used inconsistently. Given the centrality of diagnostic thresholds in the BP debate, clearer definitions are essential.

We would like to thank the reviewer for the comments. These terms have been explicitly explained.

“Recurrences during the blanking period—commonly referred to as early recurrence of atrial tachyarrhythmias (ERAT)“ – introduction section

“False positive: occurrence of early recurrence without late recurrence” – PRO section

“False negative: lack of early recurrence with occurrence of late recurrence” – PRO section

9.      Patient-Reported Outcomes (PROs)

The manuscript would benefit from a more explicit discussion of PROs, such as:
- Quality of life following early recurrence.

- Anxiety or confusion caused by conflicting interpretations of procedural success.
- Patient preference in rhythm monitoring and follow-up intervals.

Further to the reviewer’s comment we have highlighted in the discussion that “*The implications of this controversy affect also patient counseling, where clarity and consistency in defining procedural success or failure are critical to shared decision-making. Conflicting interpretations of procedural success can trigger anxiety and confusion in patients thereby impairing their quality of life*.”

Specific Comments

*       Reference Formatting: Inconsistencies in citation style are present. E.g., Page 2 alternates between "(1,3)" and superscript "3". Please standardize.

Reference formatting has been standardised throughout the revised document

*       Page 3: "Large atria" as a justification for early re-intervention is questionable. Please clarify the pathophysiological reasoning or rephrase this.

We have included “large atria” in the list of factors that define a high risk state for recurrence following catheter ablation. This lowers the threshold for considering an early re-intervention. There are data supporting that large atrial size (both diameter and volume) are independent preprocedural predictors of AF recurrence following AF ablation (Berruezo et al. Eur Heart J 2007;28:836–41. – Njoku et al. Europace 2018;20:33–42. – Costa et al. Int J Cardiol 2015;184:56–61. – Zhuang et al. Europace 2012;14:638–45.). Therefore we believe that the existing evidence support the pertinent statement.

*       Page 6: Sentence is awkwardly constructed: "A recent meta-analysis showed that nearly half of patients remained free from atrial arrhythmia…" Suggested revision: "A recent meta-analysis found that more than half of patients experienced arrhythmia recurrence within five years after a single ablation, with approximately 70% achieving freedom from recurrence after multiple procedures."

We totally agree with the reviewer and we have modified accordingly the text in the revised manuscript.

*       Page 11: The statement: "A delayed but correct diagnosis is always preferable to a fast, yet ultimately misleading, one." This holds true only if treatment delay does not compromise prognosis. Consider qualifying this statement in that context.

Further to your comment and in order to avoid confusion in the last sentence of the PRO section, we have omitted this sentence.

Reviewer #2:

Confidential comments to Author:

This timely and well-balanced controversy article explores the continued relevance of the post-ablation blanking period (BP) in atrial fibrillation (AF) management. It juxtaposes two expert viewpoints: one defending the traditional 90-day BP as a necessary buffer against premature intervention and misclassification of transient arrhythmias, and another advocating for its elimination or individualization based on emerging evidence, patient-centric outcomes, and evolving ablation technologies, particularly pulsed field ablation (PFA). The introduction contextualizes the debate within the historical rationale and shifting paradigm driven by continuous rhythm monitoring and refined lesion durability.

This article successfully raises critical questions about the utility and limitations of the traditional blanking period after AF ablation in the modern electrophysiological landscape. It serves as a valuable stimulus for clinicians, investigators, and guideline committees to revisit longstanding assumptions and strive toward more evidence-based, individualized care strategies. Future studies should aim to validate personalized approaches to BP duration, ideally incorporating real-world data and patient-reported outcomes.

Strengths:
-       Clarity and structure: the manuscript is clearly structured, offering a comprehensive introduction followed by distinct, evidence-based perspectives. It facilitates clinical reflection and encourages individualized decision-making.

-       Balanced perspectives: both pro and contra arguments are well articulated and underpinned by relevant data and citations. "Pro" presents a pragmatic defense rooted in minimizing unnecessary procedures, while "Con" emphasizes methodological integrity in clinical endpoint interpretation.
-       Current and relevant: the discussion incorporates the latest trial data (e.g., CIRCA-DOSE, PULSED-AF, SINGLE-SHOT CHAMPION) and consensus documents, making the debate highly relevant, more particularly in the current era of PFA.

-       Clinical and regulatory implications: the authors acknowledge the broader impact of BP definitions on trial design, regulatory endpoints, patient counseling, and cost-effectiveness.

We would like to thank the reviewer for the positive comments

Some points merit particular attention and discussion:

-       Scope of evidence for PFA: while both authors mention PFA, the field is still maturing. Some of the evidence cited remains observational or from secondary analyses. A more explicit acknowledge of the observational nature of much of the PFA data currently available should be provided.

We agree with the reviewer’s comment. Accodingly we have added in the revised manuscript the following: “*Given that most current data are observational or derived from secondary analyses, robust data from randomised controlled trials are needed to further shape and support pertinent recommendations*.”

-       While interesting, the 2 examples cited outside of AF (PREVAIL & ISCHEMIA) are out of the scope: the significance of the BP may differ depending on the disease, and this controversy is aiming to challenge the BP specifically for AF.

Further to the reviewer’s comment we have added a paragraph explaining why commenting on these non-AF trials is relevant to the topic discussed. The concept is to present “*examples outside of AF which illustrate the danger of including blanking periods after procedures*.” Therefore, we have explained that “*While these examples are outside of AF, they clearly show how blanking periods could bias in favor of a procedure over a comparator. Imagine our AF ablation vs drug trials without a blanking period. Surely the ablation advantage would be reduced*. “

-       Overemphasis on binary framing: the article frames the debate as a binary choice (retain vs. eliminate BP). A third, more nuanced perspective—personalizing BP duration based on recurrence timing, symptom burden, and ablation modality—could be a bit more explicitly developed.

We concur with the concept of the reviewer’s comment. In this context we have included in the manuscript that several factors influence the predictive power of early recurrences including but not limited to the timing, number and the burden of AF early recurrences. However, there are no data to support a personalised BP duration. Although conceptually attractive, a smilar paradigm would greatly hinder consistency in data reporting, comparison with historical trials and the assessment of procedural efficacy. However, we have included the potential of this “third pole” in the introduction section among the questions that need to be addressed: “*Should the blanking period be abolished altogether? Should it be tailored to the ablation technology, the timing of recurrence, or the patient's clinical profile*?“

-       Patient-reported outcomes: the debate relies heavily on recurrence metrics, with limited focus on patient-centered endpoints such as quality of life, healthcare utilization, and psychological burden, which are crucial in shared decision-making post-ablation. I would suggest to incorporating or highlighting patient-centered outcomes more directly.

We agree with the reviewer that the patient reported outcomes are important in decision making post-ablation. In the end of the introductory section we have modified the last paragraph accordingly: “*As we debate this issue, the goal remains unchanged: to provide safe, effective, and timely care for our patients taking into account not only recurrence metrics, but also patient-centered endpoints such as quality of life, healthcare utilization, and psychological burden*.”

-       Need for algorithmic guidance: clinicians would benefit from a proposed decision algorithm or practical criteria for interpreting early recurrence, and undertake decisions in light of lesion durability, comorbidities, and symptomatology. Consider a brief summary or visual aid (e.g., a flowchart or table) proposing clinical scenarios where shortening vs. maintaining BP is most appropriate. If space limitation, this Figure could replace the current ones.

We would like to thank the reviewer for the suggestion. However, we consider that we have no data to support the concept of a variable BP duration based on patient characteristics, clincal scenarios/settings or ablation energy source. In addition we are concerned that, although conceptually attractive, this paradigm would hinder consistency in data reporting, prevent comparison with historical trials and complicate the assessment of procedural efficacy. Furthermore, introducing this 'third pole' to synthesize diverging opinions would weaken the value of the original controversy, which currently stimulates readers to think critically about the need for blanking period in the current ablation landscape. We initially suggested the following flowchart, but concluded it would be better to omit it.

-       Typo line 305: "insignificant".

We have corrected the typo

Reviewer #3:

Confidential comments to Author:

This is a well written and quite informative article. I think AF burden and time for re-ablation are still controversial topics that need improvements and clarification in the manuscript.
In consistency to the answers provided to other reviewers’ comments we have adapted the revised manuscript accordingly.

Please discuss and quote for AF burden:

1: Doehner W, Boriani G, Potpara T, Blomstrom-Lundqvist C, Passman R, Sposato LA,  et al. Atrial fibrillation burden in clinical practice, research, and technology development: a clinical consensus statement of the European Society of Cardiology Council on Stroke and the European Heart Rhythm Association. Europace. 2025 Mar 5;27(3):euaf019. doi: 10.1093/europace/euaf019.
PMID: 40073206; PMCID: PMC11901050.

2: Noujaim C, Assaf A, Lim C, Feng H, Younes H, Mekhael M, et al..
Comprehensive atrial fibrillation burden and symptom reduction post-ablation:
insights from DECAAF II. Europace. 2024 May 2;26(5):euae104. doi:
10.1093/europace/euae104. PMID: 38646912; PMCID: PMC11077606.

and for recurrences please consider the following

1. Di Biase L, Reddy VY, Bahu M, Newton D, Liu CF, Sauer WH, et al. Early versus late atrial fibrillation recurrence after pulsed field ablation: insights from the admIRE trial. Europace. 2025 Feb 5;27(2):euaf007.
doi: 10.1093/europace/euaf007. PMID: 39820338

2. Onishi N, Kaitani K, Nakagawa Y, Inoue K, Kobori A, Nakazawa Y,  et al The association between late-phase early recurrence within the blanking period after atrial fibrillation catheter ablation and long-term recurrence: Insights from a large-scale multicenter study.
Int J Cardiol. 2021 Oct 15;341:39-45. doi: 10.1016/j.ijcard.2021.07.053. Epub 2021 Jul 31. PMID: 34343532.

All the abovementioned manusripts have been included as references
